# Salve for a Sore Head

**Published:** 1885-07

**Authors:** 


### SALVE FOR A SORE HEAD.

The Columbus, Ohio, *Medical Journal* attempts the role of "me too," Platt, to the *N. Y. Medical Record's* Conklin, on the International Medical Congress question—and in an abortive attempt to be smart at our expense, apparently purposely misquotes us—Listen :

"And so there was a kick. The original kick, it would appear, was kicked in Texas, whence, in the vigorous, but slightly *mixed* figure of the *Courier Record*, it (the kick) "rolled into one mighty billow, and at New Orleans inundated, swamped, aye *snowed under* the whole arrangement."

Miss Augusta Evans said (and we agree with her) "It is hard to have to furnish the ideas, and the brains, too, to comprehend them."

Our language was unmistakable to an *ordinary intelligence*, therefore it was purposely misquoted—we will be generous enough to say. We wrote :

"A kick, which, like a pebble thrown into the sea, set in motion a wave of popular indignation, which rolled into one mighty billow," etc., as waves sometimes do.

If there is anything "mixed," it must have been this editor's drinks. However, we are willing to admit he knows more about kicks than we do; they *may* raise billows, and blisters too, for all we know.

This genial (?) and would-be witty contemp(t)orary has a good deal of bile to disgorge on the subject of the action of the A. M. A. at New Orleans with regard to the committee's report, and spurts it out thus : (Quoting from the same article.)

"The committee had made no provision for the eloquent champion of Texas mothers ; they had ignored the oleates ; * * * * had not given the 'rural deestricts' their *numerical* ratio of officers ; whole states were ignored ; * * * even Ohio had but fourteen, and these her metropolis had, all but one, * * * and so it was decided to increase the size of the committee, and, otherwise so arrange matters that the ranches of Texas, the prairies of Kansas, the mines of Colorado and the forests and wheat fields of the great Northwest should all be represented among the officers of this Congress."

Why did not our colleague from Porkumbus finish the list and say, also, the "Hogs of Ohio?"

But, to continue the quotation: "Verily, we trust the original committee, and their rustic accessories, may duly exercise, the former, patience, the latter, humanity, and both, moderation, to the end that the congress may be a success; for unless they do this, we greatly apprehend the Texas kick will overturn the fat in the fire."—*Columbus Medical Journal.*

The eloquent champion of Texas mothers (*proud distinction*) is now provided for, thanks to the discriminating good sense of those "rustic accessories" from the "rural deestricts." Thanks for the title, too, to our *larded* cotemporary, who speaks so naturally about "fat." Did our readers ever observe how naturally some men incorporate into their social lives, and their conversation, the vulgarisms of their trade or calling? We once asked a shoemaker what had become of his boy, and he said he had "pegged out." A sailor cannot, even in society, avoid nautical phrases, and speaks of one's being "rigged out," etc.; while railroaders express everything in the parlance of the road; how natural then to hear this denizen of the hog-killing State speak of the overthrow of the new code scheme, as "throwing the "fat" in the fire."

He makes a greasy, but spiteful and envious allusion, also, to a distinguished author, teacher, scholar and physician, who has earned laurels for himself in a new field, and added lustre to American medicine. Such reference as "the oleates," intended for Dr. Shoemaker, though malicious, is as complimentary as the reference to ourself, as the "eloquent champion of Texas mothers." We will ever be under obligations to our unctuous colleague; and no doubt, so will Dr. Shoemaker, for the compliment to him; how-be-it expressed in language more oleaginous than elegant, and we only regret that our cotemporary is not identified with some scientific work, or some noble act, that we might return the compliment.

Verily, the new code 'fat' *is* overturned, if the clean sweep of new code names from the list, at Chicago, can be expressed in such a greasy way, and we are proud to believe the "champion of Texas mothers," and the "Texas kick", to which our cotemporary so sneeringly alludes, had much to do with it.

